# Narrow Band Imaging with Magnification Endoscopy for Celiac Disease: Results from a Prospective, Single-Center Study

**DOI:** 10.1155/2013/580526

**Published:** 2013-08-06

**Authors:** L. De Luca, L. Ricciardiello, M. B. L. Rocchi, M. T. Fabi, M. L. Bianchi, A. de Leone, S. Fiori, D. Baroncini

**Affiliations:** ^1^Gastroenterology and Digestive Endoscopy Unit, “San Salvatore” Hospital, Piazzale Cinelli, 1-61121 Pesaro, Italy; ^2^Department of Clinical Medicine, University of Bologna, Via Massarenti 9, 40138 Bologna, Italy; ^3^Department of SUAN, University of Urbino, Via Ca le Suore 2/4, 61029 Urbino, Italy

## Abstract

In celiac disease (CD), the intestinal lesions can be patchy and partial villous atrophy may elude detection at standard endoscopy (SE). Narrow Band Imaging (NBI) system in combination with a magnifying endoscope (ME) is a simple tool able to obtain targeted biopsy specimens. The aim of the study was to assess the correlation between NBI-ME and histology in CD diagnosis and to compare diagnostic accuracy between NBI-ME and SE in detecting villous abnormalities in CD. Forty-four consecutive patients with suspected CD undergoing upper gastrointestinal endoscopy have been prospectively evaluated. Utilizing both SE and NBI-ME, observed surface patterns were compared with histological results obtained from biopsy specimens using the k-Cohen agreement coefficient. NBI-ME identified partial villous atrophy in 12 patients in whom SE was normal, with sensitivity, specificity, and accuracy of 100%, 92.6%, and 95%, respectively. The overall agreement between NBI-ME and histology was significantly higher when compared with SE and histology (kappa score: 0.90 versus 0.46; *P* = 0.001) in diagnosing CD. NBI-ME could help identify partial mucosal atrophy in the routine endoscopic practice, potentially reducing the need for blind biopsies. NBI-ME was superior to SE and can reliably predict *in vivo* the villous changes of CD.

## 1. Introduction

Standard endoscopy (SE) does not usually allow the visualization of duodenal villous patterns and may be inaccurate in patients with celiac disease (CD) [1–4]. In CD the intestinal damages can have a patchy, “stain-like” distribution, and the macroscopic features can be more or less dependent on the degree/severity of the histological lesions [2]. Indeed, at SE, partial villous atrophy may elude detection, and a normal endoscopic appearance of the mucosa does not necessarily imply normal histology.

Several endoscopic features observed during SE reflect the presence of villous atrophy; however their sensitivity varies from 50% to 94% [5, 6]. Sensitivity is particularly low in patients with subclinical CD, partial villous atrophy, or patchy disease [1, 2, 7]. Improved visual identification of suspected mucosal atrophy could assist in targeting biopsies and thereby increasing the sensitivity of endoscopy [8]. Different emerging techniques have been evaluated, alone or in combination, to enhance the ability of the endoscopist to detect mucosal abnormalities, including chromoendoscopy, water-immersion techniques, magnification endoscopy (ME), alone or combined with acetic-acid instillation, and optimal band imaging [8–12].

A previous observation reported the combination of high-resolution Narrow Band Imaging (NBI) with ME (NBI-ME) to obtain targeted biopsy specimens indicating a higher simplicity (with the switch of a button) than chromoendoscopy, thus reducing the procedure time [13]. In fact, NBI-ME is a simple tool that allows detection of the subepithelial microvascular architecture and mucosal microsurface structure in a variety of pathological conditions [14, 15]. To our knowledge there is not a prospective study that addresses the performance of NBI-ME in evaluating patients with suspected CD. The aim of this study was to assess the correlation between NBI-ME and histology and to compare the diagnostic accuracy between NBI-ME and SE using histology as gold standard in detecting villous abnormalities in CD.

## 2. Materials and Methods

### 2.1. Subjects and Endoscopic Procedures

Forty-four consecutive patients (17 males, 27 females, age range 14–73 years, mean age 36.5 years) with clinical history suggestive of malabsorption (weight loss, chronic diarrhea, iron-deficiency anemia, etc.) and serologic suspicion for CD as positive or borderline antiendomysial (normal values are absent for both IgA and IgG) and antitransglutaminase antibodies (normal values 0–10 U/mL) were prospectively enrolled in the study. The presence of severe gastrointestinal or systemic disease was considered as an exclusion criterion (e.g., chronic pancreatitis, liver cirrhosis, and blood coagulation disorders). The study was performed under the local ethics committee approval.

Prior to endoscopy, a written informed consent was obtained from all participants. In order to prolong the procedure for the evaluation of the duodenal mucosa with NBI-ME, a conscious sedation with intravenous midazolam (0.05–0.1 mg/Kg) was used before undergoing upper gastrointestinal (UGI) endoscopy together with induction of gastrointestinal hypotony (with hyoscine N-butylbromide 10–20 mg i.v.).

**Figure 1:**
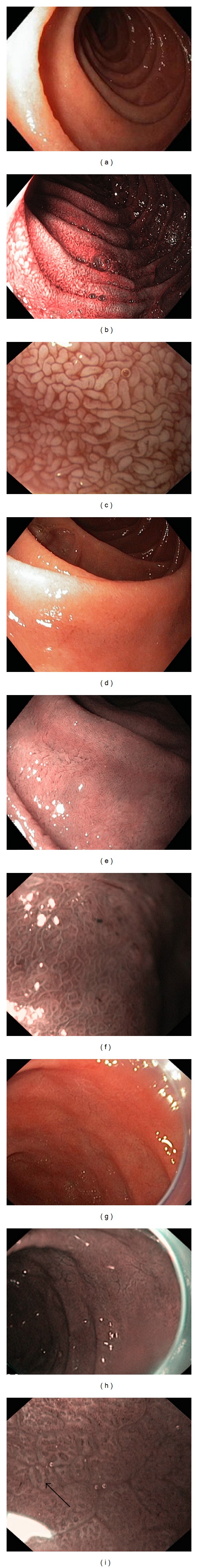
(a, b, and c) Normal villous patterns. Visualization of normal duodenal mucosa at white light SE (a). Regular villi appear well represented, thick, with a finger-like appearance at NBI system and ME (b and c). (d, e, and f) Abnormal villous patterns. Partial villous atrophy view at SE (d); NBI-ME showing a low-density of villi which appear irregular, disoriented, and with an initial pattern of surface destruction (e and f). (g, h, and i) Absent villous patterns. Marked villous atrophy with NBI system and ME (g); the surface is flat (h), “foveolar-like,” with total villi absence and wide circles (i: black arrow).

All procedures were performed with an Olympus GIFQ 160Z, Exera II (Olympus, America Corp., Melville, NY, USA), a high-resolution endoscope with adjustable image magnification to ×115 which included the NBI system. A disposable polyethylene cap was applied on the distal end of the endoscope to prevent slippage of the mucosa and to help secure a single area for focus, thus maintaining an optimum depth from the mucosa. A physician interviewed and selected the patients. All UGI endoscopies were performed by a single endoscopist who was blinded to the laboratory data of all patients. A regular inspection of the duodenum with SE was performed before switching to the NBI vision. A judgement on the villous appearance (abnormalities or not) for SE and for NBI-ME was then expressed. The various duodenal villous patterns at NBI-ME inspection were classified as follows: normal, abnormal (partial villous atrophy), or absent (marked villous atrophy) (Figure 1).

To avoid insufficient sampling, as recommended by previous investigators [16], 4 to 6 biopsy specimens were taken from the descending duodenum and from any other area of irregular appearing duodenal mucosa by using standard biopsy forceps. If no abnormality was macroscopically evident, random biopsy specimens were obtained. The histopathological evaluation was performed according to the Marsh classification, modified by Oberhuber [17]. Samples were evaluated by a pathologist who was blinded to the clinical data and the endoscopic findings.

### 2.2. Statistical Analysis

The relationship between NBI-ME and SE findings was compared with histology using the k-Cohen agreement coefficient. Sensitivity, specificity, and positive and negative predictive values (PPV-NPV) for both techniques were calculated using histology reports as the gold standard. The efficacy of NBI-ME for predicting villous abnormalities in CD was evaluated by the area under the Receiver Operating Characteristic (ROC) curve analysis. Statistical significance was established at *P* < 0.05 for all the analyses.

## 3. Results and Discussion

### 3.1. Results

A diagnosis of CD was histologically made in 17 (38.6%) of 44 enrolled patients, while the remaining 27 (61.4%) had a normal villous pattern. Among those diagnosed with CD, 7 (41%) showed endoscopic features of disease during SE.

**Figure 2:**
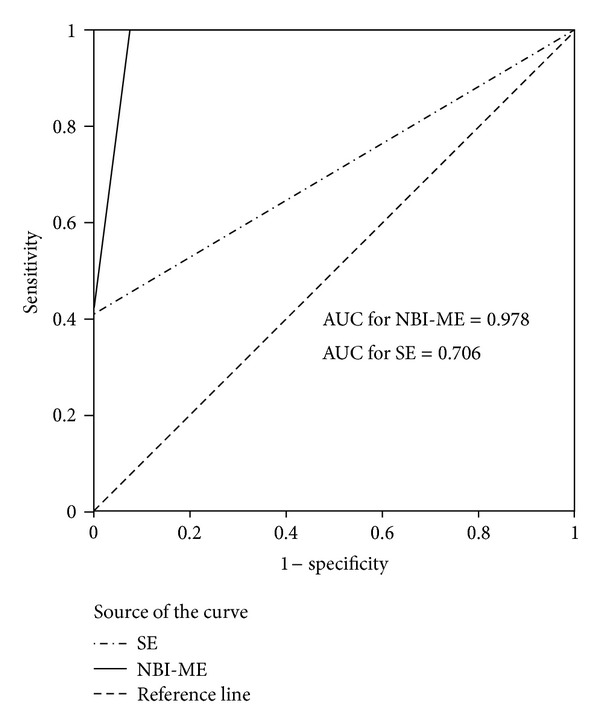
Receiver operating characteristic curves of NBI-ME and SE for diagnosing CD.

After switching to NBI-ME, a diagnosis of mucosal abnormalities was made in 19 patients. Among these, 7, which were the same as those found at SE, had a complete absent duodenal villous pattern, while 12 displayed an abnormal duodenal villous pattern. Two cases showing abnormal villous patterns at NBI-ME were subsequently classified as negative for CD at histology (Table 1). NBI-ME identified patchy areas of partial villous atrophy in 12 patients with a sensitivity, specificity, PPV, and NPV of 100%, 93%, 89%, and 100%, respectively. The corresponding values for SE were 41%, 100%, 100%, and 73%, respectively (Table 2). The area under the ROC curve for NBI-ME was 0.978 (*P* = 0.0005), indicating an excellent agreement with the histological results. The area under the ROC was 0.706 for SE (*P* = 0.023) (Figure 2). The overall agreement between NBI-ME and histology was significantly higher when compared with SE and histology (kappa score: 0.90 versus 0.46; *P* = 0.001) in determining CD (Table 3). Among 27 subjects without CD, the diagnoses were functional dyspepsia, irritable bowel syndrome, or overlap syndromes.

The mean additional time for single NBI-ME procedure was 4 minutes and 30 seconds (±1 minute).

### 3.2. Discussion

The first report on the use of ME with chromoendoscopy to highlight duodenal villous pattern was published by Siegel et al. [18] demonstrating an increased detection rate of focal villous atrophy as well as partial villous atrophy compared with SE in patients with malabsorption. However, some limitations of this technique related to difficulty in achieving a complete and an even coating of the mucosal surface with the dye [12], increased cost and procedure time, and the lack of visualization of the vascular pattern have prevented the widespread use of vital dye staining chromoendoscopy techniques. Recently a new technology called “Fuji Intelligent Color Enhancement,” a virtual chromoendoscopy, was introduced to enhance the contrast of the mucosal surface without the use of dyes, through the ability to select better spectral images decomposed from ordinary endoscopic images [19]. Cammarota et al. [12] published an original open, prospective, single-centre trial on the potential of a similar system, the optimal band imaging (OBI), for predicting the duodenal villous morphologic characteristics in patients with suspected CD. The authors concluded that the OBI system, in association with ME, allows clear visualization of the duodenal pattern, with a potential role in optimizing the diagnostic accuracy of endoscopy in CD.

In the present study we show that the NBI system, associated with ME, has provided superior performance than conventional endoscopy in detecting mucosal abnormalities on otherwise normal appearing duodenal mucosa. We found high sensitivity and specificity values with an overall agreement between NBI-ME and histology significantly higher when compared with SE and histology in determining duodenal villous pattern features. Moreover, the ROC curve analysis demonstrated that the NBI-ME performance was greater than SE, showing an excellent agreement with the histological results.

The NBI system consists of a sequential electronic endoscope system that can select better spectral images using particular luminous bands, thus enabling to filter incidence light resulting in some kind of “coloration without coloring.” Since the gastrointestinal tract is mainly composed of blood vessels and mucosa, narrow band illumination, which is strongly absorbed by hemoglobin and penetrates only the surface of tissues, is ideal for enhancing the contrast between the two.

However, our results have some limitations. First, we did not assess the inter- or intraobserver reliability of the mucosal patterns. Consequently, although duodenal villous classified patterns were normal, abnormal, or absent, we have not evaluated the correlation between partial or marked villous atrophy with histological score. In fact, our main objective was to establish the presence or absence of CD. A previous report by Badreldin et al. [10] on the potential role of zoom endoscopy for the diagnosis of the various degrees of villous atrophy showed a sensitivity of 90.7% and specificity of 63%. In their study the main disagreement between zoom endoscopy and histopathology was the distinction between normal tall villi and morphologically normal but shortened villi, which depends on the assessment of villus height. More recently another study, using a simplified classification, demonstrated the feasibility of using NBI-ME for the detection of villous atrophy in patients presenting with suspected CD [20].

Second, among the criticisms that have been raised for the use of NBI-ME was the slightly increased procedure time, including conscious sedation, for routine application. We observed an additional mean procedure time of 4 minutes and 30 seconds.

Third, as reported by other authors [12], all patients who underwent UGI endoscopy had clinical history of malabsorption or serologic suspicion for CD, thus having a high pretest probability for duodenal abnormalities.

Fourth, we have found two false-positive cases at NBI-ME which reduced the specificity. It is possible that this approach could lead to an overestimation of the findings, and the results could be influenced by the high pretest probability for duodenal disease. It is widely known that the prevalence of CD in open access endoscopy is likely to be underestimated, with missed diagnose, ranging from less than 1% to 16% [21]. The ideal diagnostic technique approach for CD should increase accuracy and sensitivity and be easy to perform, cost saving, and repeatable, and not be time consuming [22].

Furthermore, other endoscopic options, such as confocal endomicroscopy [23] and optical coherence tomography [24], that were studied in patients with suspected CD achieved good results. However, these techniques are hampered by technical problems. In particular, difficulty of image acquisition/stability, with potential distortion and artifacts, and a long learning curve would restrict their wide use on the basis of the local availability of equipment and expertise. Moreover, according to Fedeli and colleagues [25], the combination of two or more simple new endoscopic approaches, such as OBI together with ME, during water-immersion would obtain outstanding images of the villous pattern.

Using these new endoscopic techniques, the same authors have proposed an algorithm to minimize the need for duodenal biopsy in patients with suspected CD in particular for those with total villous atrophy [22], or in such circumstances that could involve patients who are on anticoagulation therapy and that cannot be safely interrupted [26]. As suggested in a correspondence [27], we believe that it is not possible, at present, to avoid biopsies in CD, both for the initial diagnosis and for followup. In fact it is not possible to make a precise differential diagnosis of *Giardia lamblia* infection or Crohn's disease (where there could also be changes of serological markers) [28] and eosinophilic jejunitis or HIV enteropathy [29]; furthermore, the histological evaluation is fundamental because it allows verifying improvement of duodenal lesions after gluten-free diet or the absence of mucosal recovery that needs an analysis of molecular markers in the suspicion of a T-cell dysplasia lymphoma [30].

## 4. Conclusions

In conclusion, our findings show that the NBI with ME represents a simple technique that could help identify patchy areas of partial mucosal atrophy and then estimate the extension, even considering frequently mixed patterns. With these tools it is also possible to predict, in a reliable manner, “minimal changes” of duodenal villi in CD occurring* in vivo* and, importantly, improve biopsy sampling by potentially reducing the need for blind biopsies and false-negative cases.

## Figures and Tables

**Table 1:** Histological diagnosis and corresponding endoscopic findings.

		Endoscopic findings	
	*n*° patients	SE	NBI + ME
CD	17	7	19^§^
Normal	27	37	25

^§^7 absent villous patterns.

12 abnormal villous patterns.: 2 negative, histology for CD.

**Table 2:** Diagnostic accuracy of SE and NBI with ME.

	Sensitivity (%)	Specificity (%)	PPV (%)	NPV (%)
NBI + ME	100	93	89	100
SE	41	100	100	73

**Table 3:** Overall agreement (k-Cohen coefficient) between endoscopic findings and histology reports.

NBI + ME and histology	0.90*
SE and histology	0.46

*P = 0.001 (Student's *t*-test).
